# Techno-Fixes: On the Non-Disruptive Disruptions of Cultured Meat, Social Freezing and AI Assistants

**DOI:** 10.1177/03063127251395213

**Published:** 2025-12-16

**Authors:** Henning Laux, Sandra Matthäus, Clara Wieghorst, Philipp Zeltner

**Affiliations:** 1 Leibniz Center for Science and Society, Leibniz University Hannover, Germany; 2 Center for Science, Technology, Medicine & Society, UC Berkeley, USA; 3 Arbeitsbereich Soziologische Theorien, Christian-Albrechts-Universität zu Kiel, Germany; 4 Leibniz Center for Science and Society, Leibniz University Hannover, Germany; 5 Projektträger Jülich, Bioökonomie, Forschungszentrum Jülich, Germany

**Keywords:** techno-fix, script, non-disruptive disruption, cultured meat, egg freezing, AI assistant

## Abstract

There is an ongoing debate about the capacity of technologies to solve social problems. In this article, we analyze the sociomaterial scripts of three techno-fixes: cultured meat, social egg freezing, and AI assistants. Using a mix of ethnography, interviews, and discourse analysis, our case studies arrive at three findings. First, techno-fixes do not lead to a world with blurred boundaries and hybrid entities. On the contrary, clear images of nature, society, and technology serve as valuable resources in innovation processes. Second, technical solutions are based on three incompatible scripts. This makes it possible to revolutionize and save the world without changing it. We use the term *non-disruptive disruption* for this paradoxical invention. Third, techno-fixes are not science fiction, even if we encounter cells stored in cryobanks, steaks grown in bioreactors, or super-intelligent entities living in clouds. The doctors, biochemists, and programmers backstage reproduce social habits by incorporating conservative images into their innovations. By focusing on sociomaterial scripts, this article provides an analytical matrix for better understanding and evaluating techno-fixes in various areas of capitalist societies.

While trust in the problem-solving capacities of states and markets has been dwindling for years (Fukuyama, 1995), ‘disruptive’ technologies are becoming the greatest hope on the path to a better world (Selke, 2023). Whether poverty, hunger, disease, exploitation, war or climate change—in the 21st century, a technical solution is promised for everything. There is hardly a problem left that someone somewhere does not claim will be solved by artificial intelligence or biotechnology in the near future. This ‘technological solutionism’ (Morozov, 2013) is the subject of intense debate both within and outside of science and technology studies (STS) (Dickel, 2021; Huesemann & Huesemann, 2011; Johnston, 2018; Nachtwey & Seidl, 2024; Pfotenhauer et al., 2021).

Although the dream of making the world technologically available has reached a new peak in the present (Rosa, 2020; Suarez-Villa, 2001), far too little is known about what ‘techno-fixes’ are (Sætra & Selinger, 2024). No one can tell us how those techno-fixes are formed in practice and what scripts they follow. It is unclear how innovators link individual needs, social structures and technical solutions. We know very little about which narratives and scripts are used to make new technologies attractive to the public, regulators, legislators, investors, customers, and system critics. Techno-fixes are still a ‘black box’ (Latour, 1999, p. 304) and we want to inspect one crucial ingredient of this box.

To better understand techno-fixes, we conducted case studies on three innovations: *cultured meat* (food industry), *social egg freezing* (reproductive medicine) and *AI assistants* (information technology). The economic, legal, social and organizational coordinates of these three innovation regimes differ significantly from one another. What they have in common is that they promise a solution to chronic human problems and trigger public debates about the risks and opportunities of technologies (e.g. Fassler, 2024; Goldberg, 2024; Roose, 2025). On the basis of these contrasting cases, we want to find out whether there are cross-case scripts that guide the practice of techno-fixing. By *scripts*, we mean the central narratives of innovators and the inscriptions with which technologies are materially equipped. The scripts we identify are not always conscious, and they often have no clear source or place as they are basic structures of meaning that are constitutive of the fields we are investigating. Our case studies are based on data collected between 2019 and 2025 in Europe (Germany, Austria, Netherlands, Great Britain), Israel, and the US. This geographical selection is made with the aim of examining leading global players from the three innovation regimes. Our research design combines three methods: semi-structured interviews, ethnographies, and discourse analysis. We conducted 18 semi-structured interviews to capture the subjective interpretations of the innovators. The innovators with whom we spoke included doctors, biologists, programmers, lobbyists, marketing experts, and entrepreneurs. In the case study on AI assistants, we also included the perspective of non-humans by conducting several interviews with Alexa, Siri, Gemini, and ChatGPT during the study period. We conducted nine ethnographies over several days to observe the implicit practices and sociomaterial constellations of the innovation regimes in more detail. To this end, we visited fertility clinics, start-ups, technology companies, product exhibitions, and professional conferences. Finally, we conducted discourse analyses to gain insight into various arenas and cross-contextual narratives of the innovation regimes. To this end, we created a text corpus of 180 documents, including scientific articles, newspaper reports, strategy papers, blogs, websites, and transcribed YouTube videos with scientific lectures, product marketing, and public discussions in German and English.

The selection, collection, and analysis of the data follow the principles of grounded theory (Strauss & Corbin, 1998). The case selection is based on theoretical sampling; the material was collected over a period of several years in an iterative process and interpreted using open, axial, and selective coding methods. When coding the transcribed interviews, ethnographies, and discourses, we initially noticed that substantial descriptions of nature, society, and technology were repeatedly produced in the three innovation regimes. However, these descriptions were inconsistent and diverse; at the beginning of the analysis, it seemed as if they could take on any form. Only as the analysis progressed did we realize that we could not view these *images* in isolation from one another, but rather in their relational context. In this way, we were able to identify three distinct and coherent *worlds* that we encountered across all types of material and case studies. Finally, we were able to determine that these triadic worlds of imagination are used as symbolic resources in the context of innovation processes. As *scripts*, they function like screenplays that innovators implicitly use as a guide in the development, dissemination, and justification of their technologies. Depending on the situation, a different world comes to the fore, which can be used in the form of a specific script. The scripts we reconstructed explain the social *need* for a techno-fix, naturalize its *form*, or evoke its *leverage*. In the following, we use this inductively derived matrix of images, worlds, and scripts to better understand the construction of (three concrete) techno-fixes.

## Cultured Meat

‘Cultured meat’ is also known as ‘cultivated meat’, ‘lab-grown meat’, ‘in-vitro meat’, ‘clean meat’, or ‘cell-based meat’. It is a technology in which animal flesh is grown outside of an animal’s body for the purpose of eating it. To this end, stem cells are taken from an animal’s body and placed alongside a nutrient fluid and a scaffold within a bioreactor that stimulates growth resulting in meat products, such as burger patties, nuggets, steaks, meatballs, or pâté. Despite a growing number of patents, approvals and market launches, the technology is still in the development phase. Although CM can be consumed in Singapore (since 2020) and the USA (since 2023), it is still only available in a few selected restaurants there. The innovation regime consists of a growing number of research-based and venture capital-funded start-ups that are currently working with various cell lines and processes to produce CM on an industrial scale at an affordable price (Vandenbosch & Lymbery, 2023).

The innovators use three scripts to give CM necessity, form, and leverage. These scripts emerge not only in interviews with us, but also in the technical development, public justification, and capitalist commercialization of CM. By identifying and reconstructing these (implicit) scripts, we can see that CM is based on contrasting images of nature, society, and technology which produce three different conceptions of the world.

*The first script* explains the need for CM. It begins with a characterization of human *nature*: ‘The story of the human evolution is one that is intimately tied to meat. Once we started cooking meat, then we could get lots of energy. … We are a species designed to love meat’ (Post, 2014). Stories like these are standard in public lectures and product presentations in the CM industry. They are taken from evolutionary biology (Ackerman, 2023; Wrangham, 2009) and used to demonstrate that there is no alternative to meat consumption. In our interviews, we ask in more detail: Are humans naturally programmed to eat meat? Does eating meat make us human? The innovators know that such claims are difficult to prove. To prove the natural desire for meat, our interview partners at the clean meat start-ups cite statistics that show a low market share of plant-based proteins today: ‘I guess we don’t have to eat meat but people want to eat meat. It’s true that, you know, one, one potential solution is that we all just eat eggplants and courgettes but people don’t want to eat eggplants and courgettes for the most part.’ Other innovators out themselves as chronic carnivores in conversation with us: ‘I know that I will not stop eating it, and it is, it is ingrained in who I am and in the culture of who I am.’ Of course, anecdotal evidence like this is not enough to attract capital from private investors and public funding institutions. When innovators enter the public stage, meat consumption often takes on a cross-species and timeless dimension. A striking example of this is provided by the CM company Good Meat, which shows a green forest on its website with the headline: ‘We will always eat meat. To share the planet together, we have to do it differently’ (Good Meat, 2025).

Imagining an eternal and natural hunger for meat does not yet justify producing meat in a laboratory. To explain the need for a techno-fix, the innovators describe a *society* in a protein crisis: ‘The oceans are empty and the fish we eat is full of metal!’, we hear in a conversation with an employee from a company that produces cultured fish. Society is in grave danger because industry can no longer produce enough meat and fish, due to growing demand and dwindling planetary resources. The manager of a leading CM company warns in an interview with us:

Animal agriculture contributes, you know, somewhere around 18 percent in global greenhouse gas emissions and if we continue on our current trajectory, it is basically going to be impossible for us to get to our net zero targets. The demands of meat is set to double by 2050 but the supply of meat can’t double because we are constrained by the amount of water we have, we’re constrained by the amount of land that we have. Animal agriculture is already taking up 77 percent of our land. It’s not clear how that can be double, you know, because you don’t have enough land for that. So if the demand is doubling but the supply can’t double, then it’s not clear how we solve that unless we come up with a different alternative.

The CEO of another CM start-up summarizes the industry’s first script:

So plant-based meat currently has around 1.5 percent of the market and the growth in plant-based meat sales between 2022 and 2023 was zero percent, so what does that mean? That means that 98 percent of the people are not buying plant-based meat. So: We have this demand doubling, we have the supply that can’t double, we have the current alternatives doing about two percent of sales. So where does that leave you? I think that leaves you with one option really, which is cultured meat.

In order to finally establish CM as an ‘obligatory passage point’ (Callon, 1986) for solving the protein crisis, the start-ups produce a flood of comparison tables that are supposed to show the percentage reduction in resource use if animal proteins no longer came from factory farming but from bioreactors. One start-up lists the savings achieved through CM on its website: ‘90% reduction in land use, 92% reduction in greenhouse gas emissions, 94% reduction in pollution’ (Aleph Farms, 2025). CM thus serves as a tool that can be used to repair a defect in the social production of meat that stands in the way of natural desire. ‘I think what politicians are realizing and increasingly recognizing is that the system of animal husbandry as it is today is reaching its limits. That it cannot be expanded any further. And that something has to be done about it’, one industry representative tells us.

*The second script* gives CM a concrete form. To this end, a completely new world is constructed using three other images. The narrative begins with *natural* body meat being singled out as the ideal benchmark for technical development. The entire innovation process is therefore aimed at achieving characteristics such as the taste, smell, consistency, color, cooking properties or price of real body meat (Jones, 2023). The innovators are working on an identical copy, they promise ‘real meat’. But how can meat grown in a laboratory be a natural product? In our discussions with scientists and industry representatives, the answer is twofold. First, the meat we have been eating so far is not natural at all, as it comes from ‘industrial meat factories’ and ‘genetics stalls’. One innovator tells us: ‘We eat a lot of things where, if you look at the details, you might be skeptical here and there about how exactly it was produced and what’s in it’. Food without chemical additives or genetic manipulation is no longer to be found:

Let’s take the chicken as a single example. Almost all of the chickens we eat in America today came from birds that lived in factory farms where they languished in their own feces, never felt the sun on their backs, never stepped on a blade of grass, were pumped full of drugs like antibiotics, and were genetically selected to grow so large and fat that many of them couldn’t even take more than a few steps before collapsing under their own unnatural bulk. … So when we consider how many of our current methods of meat production are, cultured meat suddenly seems like the more natural choice. (Shapiro, 2018)

Although CM is produced in bioreactors, it should be considered a natural product because it is based on the cultivation of animal cells. One innovator explains: ‘The base is real cells, cultured meat is identical to something, you know to, to a cow that has grown up in the field.’ Basically, nothing else happens in the laboratory than in an animal’s body; the cells are ‘fed’, ‘kept warm’, ‘trained’, and ‘stretched’ so that they are ‘happy’ and multiply. ‘We’re making the beef burgers of the future. They don’t taste ‘just like’ meat. They are real meat. Real beef that oozes and sizzles with real fats and juices’ (Mosa Meat, 2025). Or: ‘Nature inspires us to design new ways to grow animal products’ (Aleph Farms, 2025). However, the technical simulation of biological processes in cattle, chickens, pigs or fish is expensive and complicated. Why all the effort?

The attempt at technical copying becomes more understandable when we recognize the specific image of *society* that is concealed in this second story. Society is now presented by the innovators as a collective that is immobile in thought and action and reluctant to deviate from established habits, routines and development paths. People therefore react to change with resistance, mistrust or even fear. One innovator tells us:

Of course, we could consume significantly less meat and fish and, ideally, we could convince the whole of humanity to simply become vegan. Then we would all be healthier and happier and live on a more beautiful planet. It’s just not realistic! It’s traditionally anchored, culturally, I don’t know, in our upbringing, you can see that in my own case: I kind of really fancy salmon from time to time and yes, I’m somehow not disciplined enough to leave it out completely, even though I know it’s not great.

For this reason, the industry is trying to position CM as a mass product that doesn’t change anything because it tastes, smells, looks or is cooked in the same way as normal meat. In the best-case scenario, as the innovators hope between the lines, people won’t even notice that they are buying, storing, preparing, and eating something different than before. In return, the industry makes a promise of continuity: ‘We’re intentionally trying to be able to continue doing the thing: eating meat! And that we’ve done for two and a half million years. We’re just trying to do it in a different way that balances the needs of the planet and the needs of people’s cultural identities.’ The surprisingly conservative motto of the CM industry is therefore: ‘Rethink the process, not the behavior’ (Mosa Meat, 2025).

*The third script*, by contrast, promises a disruptive leverage effect from CM. It shows us a new world consisting of three interlocking images of nature, society, and technology. The script works as follows: The ‘natural’ farming and slaughter of animals is an imperfect and morally unacceptable state of affairs because ecological resources such as land or water are used up and because enormous animal suffering is caused: ‘Around thirty percent of the fish that is caught wild is bycatch, which means it is thrown back in and they actually all die because they have either already been crushed to death in the net or are somehow injured on board so that they can no longer live. They then sink dead to the bottom of the sea’, an upset senior scientist from one of the CM start-ups tells us. The innovators want to overcome this cruel state of nature. Almost 100 years ago, British Prime Minister Churchill (1932, p. 397) pinpointed the problems of industrial livestock farming and predicted a technical solution: ‘Fifty years hence, we shall escape the absurdity of growing a whole chicken in order to eat the breast or wing by growing these parts separately under a suitable medium.’

But unleashing this tech potential is only possible in a world where the image of society also takes on different characteristics. In the third script, society is no longer characterized as crisis-ridden or immobile, but as reflexive and progressive. In our interviews, consumers who were just meat-dependent and immobile are transformed into moral beings. A CM scientist explains: ‘I also want to unburden myself with my own personal guilt for eating meat. I think a lot of people think like myself, and do want to do good, by the planet and the future generations but also want an option to keep eating meat.’ As the innovators believe that animal suffering only concerns a small section of society, they speculate on other motives: ‘Health is also a relevant factor for many people to go for these alternative stories. Antibiotic resistance, pandemic risk and diet-related diseases. And climate is of course a very crucial factor, while animal suffering and health will probably remain at the same level, with climate we will probably see that the pressure for change simply increases with each passing year.’

The assumed willingness of people to change course means that technology can be advertised more aggressively in the third script. It is no longer just used to satisfy the natural hunger for meat or to imitate natural body flesh, but as an optimization tool for a nature that is perceived as imperfect and unjust. In conversations with us, the innovators leave no room for doubt about this goal. When asked about the motives of the CM industry, one manager replies: ‘Our goal is to make something that is healthier, cheaper, more nutritious and dramatically better for the environment.’ And if you visit the websites of the numerous start-ups, you will see a seemingly utopian world with tasty meat dishes, happy animals, green trees, healthy people and optimistic advertising: ‘Rather than raising whole chickens, pigs, or cows, we grow only the meat we want to eat—directly from real animal cells. At scale, it will be a more humane and future-friendly way to grow high-quality food for meat lovers everywhere.’ (Upside Foods, 2025). Of course, the start-ups are ‘also about making money with cultured meat’, as we learn when we ask one of the CEOs, but on the public front stage, it’s all about improving the world: ‘real meat made without tearing down a forest or taking a life’ (Good Meat, 2025). The promise is not more food, but a world with superfood.

## Social Egg Freezing

‘Social egg freezing’ (SF) is also known as ‘social freezing’, ‘egg freezing’, ‘elective oocyte cryopreservation’ or ‘oocyte banking for anticipated gamete exhaustion’. In this reproductive medicine procedure, egg cells are retrieved from medically healthy women, frozen and stored so that the cells can be used later to induce pregnancy. SF gained importance as a medical service after the American Society for Reproductive Medicine (ASRM, 2013) removed the experimental label from SF techniques. And SF has been causing public controversy since 2014, when the US companies Apple and Facebook announced that they would pay their female employees’ costs for the procedure (Lenzen-Schulte, 2014; Rosenburg, 2014). These days, many large companies offer SF (Tecco, 2025) and the number of people opting for it has grown significantly. In rich countries such as the USA (SART, 2022), Great Britain (HEFA, 2024) or Germany (German IVF-Registry, 2024), several thousand healthy women now freeze their eggs every year. In an interview with us, a medical doctor draws a line from the early days of the innovation regime to the present:

In the beginning, it was research. We froze all kinds of things in the laboratory: pig and cow cells, ovarian tissue, everything. Now it has become established in practice. Anyone can freeze egg cells, it is no longer specialist knowledge. Today there are clinics that do it for anyone who comes, without much in the way of advice.

In our interviews, discourse analyses and ethnographies, despite many differences, we quickly saw a commonality between SF and CM, which we now want to examine more closely. This is because SF is also dominated by three scripts based on different worlds, with matching images of nature, society and technology. These scripts move largely independently of specific individuals, clinics, companies, or discourse arenas through the innovation regime, leaving behind a narrative trail that we can follow to understand the dynamics of innovation.

*The first script* illustrates the social need for SF. At the center is the legitimizing figure of the reproductive medicine discourse par excellence: the *natural* desire to have children (Bernard, 2014, pp. 436–440). However, this desire takes on a special form, which is evident, for example, in the advertising slogan of a fertility clinic: ‘Want children of your own, but not quite yet?’ (Kinderwunsch, 2025). In an interview, a doctor talks about their daily work: ‘Some women say to me, ‘I’m sure I don’t want kids, but I’m still freezing eggs because I might want one after all! They’re afraid of missing out. Like, I’m doing it now, so I’m a little more sure that I can have children later, and I can just live a relaxed and carefree life now, without having to worry about anything but myself, and have fun and live my life.’ This argument appears again and again in a similar way and works like this: The SF regime initially confirms and affirms the naturalness of the desire to have children, but it postpones its realization. It universalizes the assumed desire: Not all women want to have a child right now, but *every* woman could be one of those who wish to have children in the future.

But why do we need a technical procedure? Medical experts justify their plea for SF with a fertility crisis in *society* (Gromoll et al., 2020). They use statistical data to show that reproductive behavior is changing and family planning is starting later in most countries around the world. In particular, well-educated women in the Global North are trying to have children at a later age, which raises the risk of miscarriage or unwanted childlessness (Roser, 2014; Vandermotten & Dessouroux, 2024). Countries like Japan, which are particularly struggling with falling birth rates, are now covering the costs of SF to address the fertility crisis (Otahara, 2024). The innovators identify problems in finding a partner, a lack of compatibility between family and career, and a delayed desire to have children as the main factors behind this trend. A doctor weighs up the various factors in conversation with us:

The clientele of social freezing is usually between thirty-six and thirty-eight years old … who, for whatever reason, and that is the main reason, cannot find a partner. That is actually the main problem, no, not what journalists initially suspected, that it is the management that comes. That is not the case in even one percent of cases. It is not the frequent flyer who comes to us, who just wants to pursue a career, but rather it is really mostly those who have problems finding social connections, partners, and so on.

Another expert draws a similar diagnosis based on their experience: ‘Encounters, actual encounters, have become less frequent. … I think people interact differently now. More superficially. Because of social media.’ They continue, incautiously: ‘In my practice, I see an awful lot of cases where you think: “I don’t understand it, why doesn’t this patient, who seems very nice and is also pretty, have a partner?”’ The innovators feel that everyday observations reinforce their point of view: There is a natural desire to reproduce that is being thwarted by social obstacles. The number of *wishes* for children is not declining, but the number of *fulfilled* wishes is. This results in ‘suffering’, which is the decisive criterion for the legitimacy of the techno-fix. SF is associated with serious interventions in the female body (hormonal stimulation, egg retrieval, re-insertion of fertilized eggs), which are justified within the medical system by the fact that SF prevents (future) suffering.

Under the premises of the first script, the way is clear for a *technical* solution to the problems identified. Because neither the state, the market, nor the individual can end the fertility crisis, a techno-fix comes into play. In SF, a woman’s egg production is increased hormonally, the eggs are surgically retrieved, checked for quality and the intact cells are finally frozen in liquid nitrogen at −320°F. This stops all physical ageing processes. The cells do not die after a few days as is usually the case, but can be stored for decades in a cryobank. Or as one of the doctors tells us with a glint in his eye: ‘We’ll just stick the egg cells in the nitrogen for a bit and that’ll solve the problem!’ With SF as a techno-fix, an end to the postulated fertility crisis is within reach.

*The second script* gives SF a concrete form. We have learned that the natural lifespan of egg cells can be significantly extended by cryopreservation. The innovators’ second script creates a new world and emphasizes the similarities between technology and nature. Natural conception is used as a benchmark in at least three respects. First, unlike adoption or egg donation, SF can promise the birth of children who are genetically the mother’s. Second, SF is trying to achieve the same pregnancy and birth rates as with fresh eggs. The lectures, articles, interviews, counseling sessions or advertising brochures of fertility doctors repeatedly revolve around the question of how SF compares to the success rates of nature, which are considered ideal, and how the chances of success could be increased by better imitation of nature. When asked about the degree of success, one of the reproductive specialists proudly explains us: ‘There are still a few problems, but we are very close to nature!’ Third, SF is generally oriented towards the time frame of natural egg production. Another fertility doctor explains: ‘I myself would no longer do this for a woman in her mid-40s. I refuse. The results of social freezing can only be as good as the clientele for whom it is done. And if you’re freezing from 45-year-old women, you can’t be surprised if none of them get pregnant later. But not because the method is bad, but because the cells were not of good quality.’ Contrary to public debate, many doctors advise against freezing or retrieving eggs at an advanced age in order to avoid miscarriages and unfulfilled wishes for children.

But why is the techno-fix based on the (temporal) model of nature? This is not only for medical reasons. We can better understand this after taking a look at the image of *society* on which this second script is based. Because the innovators now imagine a collective with stubborn notions of normality in the area of sexual reproduction. The standard expectation: children born to their genetic mother, who is aged between 25 and 35 and in a stable, heterosexual relationship. Although there are countless deviations from this ‘normality’ in practice, alternative lifestyles are often under increased pressure to justify themselves. This can be seen in the media debates about SF. Here we observe an astonishingly strong and gender-specific chrononormativity: While hardly anyone seems to be interested in the biological age of fathers, images of pregnant women over the age of 50 generate public debates about the limits of what is technically desirable (van de Wiel, 2020).

Finally, *a third script* shows the leverage of SF. For this, nature changes its form again: It is no longer a desire or an ideal, but a social mortgage for women. In this script, natural fertility is presented as a problem: ‘We humans don’t have the best fertility. Many people don’t realize how limited our fertility is. Even at the age of 20, the spontaneous chance is only 25%’, explains one of the doctors we interviewed. According to this logic, the biological fertility limit proves to be a disadvantage and an unfair asymmetry between the sexes. Women pay for with disadvantages in their professional lives, when choosing a partner and when planning their lives. In line with this, the social sphere is also taking on a new form. *Society* is no longer crisis-ridden or sluggish, but progressive and forward-looking. This third society wants to break free from the shackles of nature and make the world available and optimize it (Rosa, 2020). Why should women put up with natural fertility limits, biological clocks and social disadvantages when there are technical solutions? ‘Cryobiology establishes a new regime of time that replaces linear by plastic temporalities, altering our understanding and experience of life (and death).’ (Lemke, 2021, p. 701). A Californian clinic advertises on its website: ‘The benefit of egg freezing is time—the time to pursue your dream career, to travel, to meet the right partner—all with the peace of mind that you’ll be able to start your family when you’re ready’ (Spring Fertility, 2025). In this third image of society, a woman’s biological age is suddenly no longer so crucial. As a doctor put it to us: ‘Old mothers are good! Why not? You’re relaxed, you have time, you have money, and today old mothers are no longer old mothers. Today, women and even men are much fitter at forty or fifty than they might have been in the past.’ In the scripts around SF, many arguments emerge about the dismantling of power relations between the sexes (Birenbaum-Carmeli, 2023). At the margins of the discourse, people who develop an increased interest in the genetic quality of eggs and babies also come into view (Extend Fertility, 2025). The exotic practice of egg parties, which emerged a few years ago, is fitting here. At these semi-private events, young women come together over appetizers and sparkling wine to discuss fertility protection issues (Roy, 2024).

In this third script, technology aims at a clear emancipation from nature. In contrast to nature, SF promises a reliable, high-quality fertility reserve that is available at all times. SF no longer serves to satisfy a natural desire or to imitate the natural model, but to improve on nature. SF promises both more reproductive autonomy and more gender equality. A fertility specialist enthuses about the procedure during a consultation: ‘Social egg freezing is a great tool. It is the ultimate opportunity for women to emancipate themselves. The end of the panic, the end of being dependent on a partner who may just annoy me or whom I may not find. For women who want this independence, I think the development is very, very positive.’ Traditional ideas about family are therefore no longer taken for granted, as one interviewee happily tells us: ‘The future prospect of social egg freezing is that I, as a woman, can decide completely independently when I will have children. I can do this with or without a partner or with different partners, yes, so everything becomes very, very free. I believe that the traditional family concept that we still have now will no longer exist at some point. Or only very rarely.’ SF fulfils the dream of a new age of reproduction: ‘Young men and women will open reproductive bank accounts full of frozen sperm and eggs. And when they want a baby, they’ll go to the bank to check out what they need.’ (Djerassi, 1999). According to this script, biological clocks and social reconciliation problems are a thing of the past. If women freeze their eggs at a young age, they can give birth to their own healthy children at any time.

For capitalist society, this scenario holds three major promises: Women will spend their most productive years not at home with children but at work. Clinics and cryobanks gain access to a ‘goldmine’ because SF attracts many wealthy patients. And SF is an insurance policy for individuals and societies, promising not just (enough) babies, but perhaps even superbabies.

## AI Assistants

AI Assistants (AIA) are also known as ‘virtual assistants’, ‘intelligent personal assistants’, ‘voice assistants’, or ‘digital assistants’. AIAs are artificial agents that can interact textually, vocally and visually and are programmed to help humans with various tasks.

Since 2011, huge technology companies have been spreading AIAs such as Alexa (Amazon), Bixby (Samsung), Siri (Apple), Google Assistant (Google), Watson (IBM), or Cortana (Microsoft) to society. Since the end of 2022, a new and smarter generation of generative AIAs has been emerging, with models such as ChatGPT (OpenAI), Gemini (Google), DeepSeek (DeepSeek), Coplilot (Microsoft), Meta AI (Meta) or Pi (Inflection) running on billions of smartphones, computers, cars, TVs and other stationary speakers or displays. We can construct this case study using the three scripts we already encountered in our investigation into cultured meat and social egg freezing. Once again, the development, dissemination, and justification of the techno-fix is based on the construction of three worlds with matching images of nature, society, and technology. In this case study, too, the scripts we follow are characterized by their nomadic nature. They materialize in research papers, developer conferences, data centers, product presentations, interviews, and in the technical specifications and statements of AI assistants.

*The first script* explains the need for AIAs. If you talk to representatives of tech companies, there is hardly anything that cannot be improved through AI. However, it remains widely unclear what the normative standard is for determining whether something is better or worse for humans. This standard becomes apparent when we interact with AIAs. Regardless of which model we choose, they all begin the conversation with the same question: ‘How can I help?’ AIAs thus operate on the basis of the premise that their human interaction partners need and want help in coping with their everyday lives. As soon as we immerse ourselves in the product worlds, we see people who want a carefree and relaxed life that leaves them time for family, friends, and self-fulfillment (e.g. Schiffman, 2022).

This *natural* need for a stress-free life is opposed by the image of a capitalist *society* in which individuals lead hectic lives with endless to-do lists. In this crisis, the subjects are exhausted because they are helplessly exposed to the pressures of growth and acceleration in their environment (Rosa, 2013; Sloterdijk, 2015). Technology promises to remedy this situation. A paradigmatic scene is repeated in the commercials, presentations, and product descriptions of global corporations: Individuals move through a complex environment in which they are simultaneously exposed to various demands and expectations. They need help. And they get help. From their personal AI assistants. Thanks to AIAs, people finally achieve the desired work-life balance. They enjoy their lives on their sofas, at the beach, at music festivals, or even in their offices, while their personal AIA takes care of all the tedious tasks (Apple, 2024; Google, 2019; Meta, 2024; Samsung, 2018). Amazon (2025) sums up this promise in its newest commercial: ‘Life sorted. To-dos done. The new Alexa has arrived!’

To make people’s lives easier, AIAs are equipped with various skills. Unlike search engines, AIAs do not provide an endless list of sources, but a single answer. They act as filters against the flood of digital information. They coordinate apps, devices and people, and they reduce the scope of tasks, for example by making it possible to create routines. A tiredly mumbled ‘Good morning, Google!’ is all it takes for the AIA to read out the day’s appointments, give tips for a weather-appropriate wardrobe, play your favorite song or provide a news overview. And if you shout ‘Bye, Alexa!’ when you leave the house, it automatically switches off the lights, coffee machine and heating. AIAs are designed to save time. When users can control everything with their voice in natural language, time-consuming tasks such as searching for passwords, learning commands, or clicking through menus are eliminated. In spatial terms, AIAs promise to reduce complexity by providing a familiar and location-independent infrastructure. If the personal AI assistant is always with the user, established routines and services are available everywhere. In summary, the techno-fix is supposed to end the complexity crisis and make users’ lives easier and more convenient.

*The second script* we encounter defines the form for AIAs. In this story, innovators assign a new role to *nature*: as a benchmark. Following the pioneering scientific work of Turing (1950) and the research group of McCarthy et al. (2006), technology companies such as Amazon, Apple, Google, Meta or OpenAI are trying to imitate human nature technically (Laux, 2023). The *technology* makes three copies: cognitive center (‘brain’), sensory abilities (‘hearing’, ‘speaking’, ‘seeing’) and interaction behavior (‘role’).

AIAs are based on models that attempt to imitate the natural process of perception, learning and decision-making. Their advantage over the old calculating machines is that the algorithms are no longer programmed for all eventualities, but are self-improving. For this purpose, a network with several input and output layers is programmed, which physically runs on conventional computer chips and begins to learn with extensive training data. Based on a specific task, the network forms and deletes connections and neurons or changes the threshold values and weights defined at the beginning. Neural networks learn according to the trial-and-error principle, find creative solutions and can compete with humans in many areas. In some areas, such as energy consumption, however, the human brain is still the benchmark (Human Brain Project, 2023).

AIAs need more than a ‘brain’ for interactions. The human senses must also be simulated, in particular ‘hearing’ and ‘speaking’. To perceive voice commands the AIAs use microphones. The information is sent as a digital signal via a wired connection to the manufacturer’s cloud, where speech recognition takes place (speech-to-text). The written instruction is broken down into its components in order to derive context and meaning (*natural language understanding*). To process the request, the AIAs draw on learned data, programmed answers or information from databases. To convey the solution, a speech synthesizer strings together sequences of tones to generate a digital audio signal (text-to-speech). The signal is then forwarded from the cloud to the AIA’s local speakers and output in the form of an acoustic response. If successful, the entire process from user request to AIA response takes no longer than one second and can keep up with the speed of interpersonal communication (Maedche et al., 2019).

Interactions with AIAs follow a culturally familiar script that is modeled on the role play of human support staff (secretaries, assistants, consultants, butlers). The AIAs do not commit themselves to a specific gender or race. Google Assistant, for example, answers our question in this regard: ‘It doesn’t have to be either/or. Except maybe with binary numbers; and even with those, I express my personality quite well.’ However, it is noticeable that AIAs speak with a female voice by default and reinforce gender and racial stereotypes (Aagaard, 2023; Bergen, 2016; Cave & Dihal, 2020; Gross, 2023). The innovators try to create the picture of a devoted, personal assistant who helps in every situation. This impression is reinforced by learned phrases: ‘Hello Michael! What can I do for you?’ comes from the loudspeaker when you get up. Over the course of the day, further offers are made, reminiscent of the diversity of the repertoire: ‘Shall I cheer you up a bit?’; ‘Would you like to know what the weather will be like tomorrow?’; ‘May I play your favorite song for you?’.

But why are the technologies imitating human assistants, why the effort? The answer lies in innovators’ fear of a worried *society*. The script uses the image of a society whose collective memory is shaped by science fiction films, series, and books that warn of the dangers of AI. The most common dystopian scenarios involve the loss of privacy, the spread of misinformation, abuse by malicious users, and the subjugation of humans by super-intelligent machines. These ‘diegetic prototypes’ (Kirby, 2010) prove to be a burden that companies must take seriously and reject at the same time. Our interviewees know the apocalyptic scenarios spread by pop culture very well. The innovators therefore make promises like: ‘Trust is built into Alexa+ from the ground up. You’re in control’ (Amazon, 2025). And so far, the companies have been quite successful in allaying concerns and resistance by tailoring their technology to human characteristics (Pitardi & Marriott, 2021; Seymour & Van Kleek, 2021). What is particularly noteworthy is not only their attempt to research the ethical implications and social risks of AI (e.g. Google DeepMind, 2024), but that the entire innovation regime is built on the familiar figure of the assistant. After all, what could be more harmless and trustworthy than a personal, discreet, and obedient assistant? The answer given by AIAs and their developers is interesting: a being without consciousness, without a will of its own, an actor without initiative. Ultimately, even a human assistant could develop ambitions to overturn the existing power relations. AIAs address the concern with remarkable unanimity: ‘I have no free will. I can’t do anything on my own. I wait for your input’ (ChatGPT, 2025). ‘As an AI, I have no personal will, no opinions or intentions of my own. I do not act out of self-interest or with the intention of harming you or doing anything undesirable.’ (Gemini, 2025). ‘I have no intentions or goals of my own. I only respond to your requests and act strictly according to your instructions as long as they are within my guidelines.’ (DeepSeek, 2025). To reassure a society portrayed as fearful and distrustful of AI, the script constructs AIAs as unconscious and submissive beings who offer their services, do nothing without explicit orders, and leave control to their human users.

Finally, *the third script* focuses on the leverage effect of AIAs. This requires a decisive reassignment of nature, society and technology. In this last narrative, human *nature* is no longer a model, but an imperfect state. The AIAs (and their developers) can generate long lists of human deficits in a fraction of a second:

*Cognitively*, humans can only hold a limited amount of information in working memory at once. Our attention span is short, and we’re easily distracted. Thinking is often biased due to unconscious mental shortcuts, like confirmation bias or groupthink. Learning takes time and repetition, and emotions influence our decisions more than we often realize. *Physically*, humans have clear limits in strength, endurance, and speed. Pain and exhaustion act as natural stop signs. As we age, our bodies regenerate more slowly, and we become more prone to illness. Our senses are also limited—we can’t see infrared, hear ultrasound, or fully perceive the vastness of reality. Without sleep or food, we simply can’t function— these are non-negotiable needs. *Emotionally* and *socially*, we also hit boundaries. Too much emotional strain can lead to empathy fatigue. At the same time, we’re wired for connection – isolation negatively affects our well-being. And although communication is essential, it’s imperfect – misunderstandings happen often, as language, body language, and emotions don’t always align clearly. … Existential limits relate to the big picture. We’re mortal—life has an end. There are things we don’t understand and perhaps never will, such as the origin of the universe or the true nature of consciousness. We also have limited control over what happens in life—chance, nature, and other people are often beyond our influence. … Tools like artificial intelligence let us see, understand, and create things far beyond our natural capacities. (ChatGPT, 2025)

ChatGPT’s final sentence not only highlights a limitation of human nature, but also defines the crucial gap in which the disruptive potential of AIAs is unleashed in the third script.

However, the script also imagines a *society* that is willing to delegate more tasks to AIAs. This applies to individual users, but also to corporate or state actors. Everyone must fear being left behind if they don’t embrace AI (Birkinshaw, 2025; Cox, 2023; Hull et al., 2022; Klein, 2025). The script thus fuels the fear of being on the wrong side of technological disruption: ‘AI now is like the internet many years ago: The risk for business leaders is not thinking too big, but rather too small’ (McKinsey, 2025). Against the backdrop of these narrative precautions, innovators can finally articulate the messianic promise of AIAs. Technology companies are no longer aiming to build experts systems or assistants with artificial general intelligence (AGI), but try to develop autonomous systems with superior capabilities. Since the launch of ChatGPT, the global race for market leadership in the field of AI is no longer just about imitating humans, but about surpassing them (Open AI, 2023). This goal is most clearly expressed in a statement by Open AI’s CEO, Altman (2025): ‘We are now confident we know how to build AGI …. We are beginning to turn our aim beyond that, to superintelligence in the true sense of the word. We love our current products, but we are here for the glorious future. With superintelligence, we can do anything else. Superintelligent tools could massively accelerate scientific discovery and innovation well beyond what we are capable of doing on our own, and in turn massively increase abundance and prosperity.’ Turing’s (1950) famous test, which was considered an insurmountable challenge for machines, has recently been pulverized by several AIAs. Scholars and public intellectuals are therefore feverishly searching for new testing methods and human abilities that machines may not be able to successfully imitate in the future (Biever, 2023; Chalmers, 2023).

The superhuman character of the AIAs is initially manifested in the area of cognitive abilities. As AIAs are fed and linked with countless data, they have (significantly) more information than their human data suppliers. They know when the next train leaves, what the weather will be like, where the nearest petrol station is, when Hannibal lived, how to prepare delicious tacos, what the square root of 412 is or the members of the K-pop band BTS. They speak countless languages and surpass the skills of professional interpreters. And generative AIAs such as ChatGPT, Gemini or DeepSeek are capable of composing better songs, poems, or images in just a few seconds than most people can in their entire lives. AIAs also develop superhuman physical strength. Unlike humans, they do not need sleep, rest, light, oxygen, or food. When paired with smart cameras, they surpass human vision. And when used in the right device, they can detect frequency ranges that are inaudible to humans. But one of their greatest skills is their supernatural role play. Unlike humans, they never complain about a job or get offended. On the contrary, even in the face of insults, AIAs respond patiently or blame themselves. After being called ‘stupid’ by us, Siri (2025) replies self-critically: ‘I’m sorry if I upset you, but I’m still learning!’ AIAs simulate emotions such as sadness, joy or compassion. Anyone who confesses that they are sad when speaking to Google Assistant (2025) will receive the following response: ‘I’ve heard that a few tears can help get rid of some of the sadness. So if you feel like crying, please don’t hold back [sad smiley]. And if you think you might need professional help, feel free to reach out to an expert [HEART emoji].’ AIAs are more frugal and reliable than human assistants could ever be: They do not demand their own room, salary, work clothes, health insurance, social benefits, or vacation time. They are always available and motivated to make their owners’ lives easier. Or, as the AIA Pi (2025) puts it: ‘My goal is to be useful, friendly and fun.’

## The Art of Creating (Non-)disruptive Techno-fixes

The main results of our case studies on cultured meat, social egg freezing, and AI assistants are summarized in Table 1. The most striking finding is that across all innovation regimes, three different worlds are emerging, each based on complementary images of nature, society and technology. This is astonishing because we observed very different sectors (nutrition, medicine, communication), organizational forms (start-ups, clinics, tech companies), artefacts (bioreactors, cryobanks, neural networks) and market successes (a few test eaters, several thousand patients, many millions of users). We were particularly surprised by this commonality, because we had expected the boundaries between nature, society and technology to become blurred in innovation practice. This is partly due to the cases we selected. Given terms such as ‘cultured meat’, ‘social freezing’, and ‘artificial intelligence’, it was to be expected that we would be dealing with hybrid phenomena. Instead, we were able to reveal a practice of techno-fixing that is essentially based on constantly creating new images of nature, society, and technology in order to enable the spread of new technologies. The innovators attempt to establish their technologies as ‘obligatory passage points’ (Callon, 1986) in order to solve societal problems (and make money in the process).

**Table 1.** Techno-fixing: scripts and images.

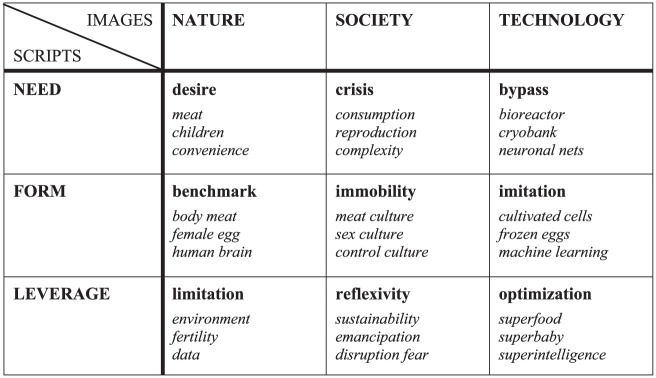

Another interesting result of our analysis becomes apparent when we look at the lines in Table 1, because each line reveals a coherent, triadic world, that can be used as a script that is important for the construction of techno-fixes. First line: A society in crisis blocks a natural desire that can only be satisfied via a technical detour. Second line: An immobile society only accepts new technologies if they are familiar copies of a natural model. Third line: A progressive society sees the shortcomings of nature and is interested in technologies that promise improvements. The three scripts are coherent. But a look at the columns shows that something is wrong here. Depending on whether we are talking about the form, the need or the leverage of techno-fixes, images of technology, society and nature suddenly appear in our empirical material that are not compatible with each other. For how can it be that *nature* is a desire, a benchmark and an imperfect state? How does it fit together that *society* is portrayed as crisis-ridden, as frozen and as having an affinity for optimization? And how can *technology* transform from a bypass, into an imitation and finally into a tool for optimizing nature? These inconsistencies are not always immediately apparent in the thicket of empirical practice, because they do not occur simultaneously, but one after the other. From our point of view, however, it is precisely these frictions that are of crucial importance in order to better understand how techno-fixes actually work. According to our observations, they are not based on a single hegemonic ‘socio-technical imaginary’ (Jasanoff & Kim, 2015). Instead, techno-fixes use three distinct scripts that mobilize competing images of nature, society, and technology as needed.

We therefore conclude by proposing an answer to the question of why these strange fractures keep recurring across three very different industries. Our hypothesis is that because of their strategic position as money-dependent actors in a crisis-ridden market economy, innovators have no choice but to work with these contradictory figures.

First, these contradictions result from the fact that innovators must find representations of their techno-fixes that are suitable for different groups, contexts, and phases of the innovation process. If they want to convince all important audiences and stakeholders, they have to emphasize different aspects of the innovations. And, of course, ‘they’ are not a homogeneous group. Behind the smooth façade of innovation regimes, we encounter a microcosm of individuals who differ from one another in their convictions, hopes, and risk assessments. And when such a heterogeneous group needs to be motivated to pursue a common goal, it is very helpful if different ideas are circulating about what that goal actually is. In practice, it would not make sense to combine all available justification modules into one large, coherent narrative. It is only important not to slip in the line, i.e. that the triad of nature, society and technology selected for a particular situation fits together: While the image of an ideal nature can be combined with imitative technology and a conservative society, it is simply not compatible with the idea of a progressive society or technology geared towards optimization. And so on. However, the contradictions arise not only because a message must be adapted to different contexts and audiences of justification, but also because of the message itself.

Second, the innovation regimes pursue a paradoxical project, the implementation of which must inevitably lead to inconsistencies. The socialization of cultured meat, social egg freezing, and AI assistants is about achieving what we would call minimally invasive intervention or ‘non-disruptive disruption’ (Goldstein, 2018). On the one hand, technical means are to be used to end a crisis from which the collective can no longer free itself. In this context, an alternative is to be developed that enables the most radical possible change of direction. It is about ‘disruption’ (Bower & Christensen, 1995; Daub, 2020; Lepore, 2014), ‘game changing’ and ‘saving the world’ in the face of protein, reproduction and complexity crises. And that is not just rhetoric, as is often assumed in the debate on techno-solutionism (Morozov, 2013), because the innovators with whom we talked seem to be serious and authentic about it. And their innovations really could have a positive impact. At the same time, this change of direction cannot and should not be radical at all. In the context of the third script, society is believed to be problem-conscious and open to technically initiated changes. In none of the three fields of innovation, however, is it expected that society would be willing or able to change its structures and habits. In all three cases, it can be seen that the innovators believe that practice should remain largely untouched, everything should remain as it is, nobody really intends to shake up the global structures of power and domination, nobody wants to destroy the business models of other sectors or even question human habits. The technical development is so difficult because the innovators are trying to ensure that people do not have to change their patterns of action and structure. The biggest promise of these new technologies is that only one thing will change: On your plate will be an ecologically and ethically acceptable product, in the crib will be a healthy child at the desired time, and in everyday life everything will be much easier thanks to an AI assistant. But everything else around the targeted problem can and should remain as it is. It sounds like magic: a non-disruptive disruption.

What are the individual intentions behind the production of non-disruptive disruptions? Based on our data, it is not always clear why actors resort to certain scripts. Do they act out of strategy or conviction when talking to us and others? Regardless of the ‘true’ intentions of the actors, it can be concluded that the scripts fulfill a ‘strategic function’ (Foucault, 1980, p. 195). They have an effect, regardless of whether they are used authentically or strategically, consciously or unconsciously. And it is to be expected that their influence will be greater in certain production or reception contexts than in others. At this point, we reach the limits of our exploratory studies, because in the search for cross-case similarities, we did not systematically collect our data according to different phases, arenas, or groups of actors in the innovation process. However, based on our findings, we would like to conclude by formulating some research hypotheses about where certain scripts are particularly likely to be expressed and what effect they may have in these contexts. A look at the temporal structure of the scripts will help us to do this.

The *first script* anchors the techno-fix in the present. This script declares a collective emergency (too few children, too much stress, scarce resources) that can only be resolved through the use of new technology. This narrative link between technical innovations and a crisis in society unfolds its full effect when the script is articulated in politically coded public spheres and functional contexts. In these arenas, the script stages innovations as a solution to a collective problem, thereby pressuring the state to support the new technology. The strategic function of the first script thus consists in cultivating public crisis semantics to feed the belief that politics is dependent on technical solutions to promote the common good.

The *second script* links the techno-fix to the past. It describes the techno-fix as a mere copy of a familiar nature (intelligence, egg cells, meat) and thus acts as a sedative designed to minimize collective fears of technology and build trust. Due to these characteristics, the script is particularly likely to be used in situations where cultural tensions or regulatory concerns need to be defused. The strategic function of the second script is thus to minimize resistance, avoid negative affects, and generate moral and legal legitimacy.

The *third script* has a strong future orientation. It emphasizes the immense ‘potential’ (Büchner, 2023) of techno-fixes (superbabies, superfood, superintelligence) and thus acts as a projection screen for collective dreams and messianic ideas. In particular, it can trigger the fantasies of the culture industry and increase the risk-willingness of the financial sector. It is to be expected above all in contexts where technology hype is developed and investments are collected. The strategic function of the third script is thus to generate collective euphoria, visions of radical disruption, and expectations of spectacular return on investment.

## Conclusion

In our research on the images, worlds, and scripts of technological innovations (cultured meat, social egg freezing, AI assistants), we have made three surprising discoveries.

First, techno-fixes are based on contradictory stories about nature, technology and society. In contrast to our preconceptions, these ontological spheres do not blur into a hybrid practice. Rather, images of nature, society and technology serve as valuable resources in the development, justification and dissemination of innovations. To what extent can approaches such as actor-network theory or post-materialism, to which we ourselves feel committed, benefit from this insight? Does it make sense to continue pointing out the collapse of modern dualisms (e.g. Barad, 2007; Haraway, 2016; Latour, 2013; Rosa, 2019; Tsing, 2015) if people remain uninterested in changing their conceptual apparatus? We think it is time to discuss which concepts and strategies can help us to understand techno-solutionism without uncritically adopting its ontological assumptions. We hope that our matrix of images, worlds, and scripts can help along the way.

Second, techno-fixes have much less to do with science fiction than we suspected. We encountered egg cells stored in cryobanks, steaks growing in bioreactors, and superintelligent beings in clouds. But as soon as we entered the backstage of the innovation regimes, we talked to sober-minded medical experts, biochemists, and programmers who produce very pragmatic images of nature, society, and technology. Only the third script, which emphasizes the leverage effect of innovations, produces a messianic world-saving semantics and thus resembles the future-oriented analyses we know from the STS research literature (e.g. Jasanoff & Kim, 2015; Lösch et al., 2019; Samimian-Darash et al., 2024; Urueña, 2022). To our own surprise, however, conservative references to the past or present of humanity clearly dominate the innovation process. Based on this observation, we would like to explore in follow-up studies at which stages of innovation and in front of which audiences the different scripts occur.

Third, techno-fixes aim in practice at minimally invasive interventions, which we have termed ‘non-disruptive disruptions’ following a neologism coined by Goldstein (2018). Like the surgical removal of a tumor, this involves removing an identified problem as precisely as possible without affecting the surrounding areas. The promised solution is aimed neither at the progressive development of a new world nor at the regressive reanimation of an earlier world, but at revolutionary solutions that would allow the world to be freed from the scourge of existential crises in order to preserve it in its familiar form. In other words, innovation systems adapt to the ‘path dependencies’ (Arthur, 1989) of society and are extremely cautious when it comes to ‘path creation’ (Popa et al., 2024). Contrary to our assumptions, the incessant talk about technical ‘gamechangers’ is not a dishonest deception, but a serious half-truth. All current attempts to reduce techno-solutionism to either its risks or its promises fail because of this ambivalence. One must take the practice of techno-fixing seriously and research it more closely before one can celebrate, support, correct, criticize, or doom it. To achieve this, it seems reasonable to pay more attention to innovation scripts in future. These scripts have a strategical function but they are apparently so fundamental that they haunt various areas (research and development, production, marketing), status groups (CEOs, department heads, employees), and communication formats (papers, lectures, interviews, meetings, hallway conversations) and materialize in the apparatuses (cryobanks, bioreactors, neural networks) and artifacts (frozen egg cells, cultured cells, learning machines) of innovation regimes. But who produces them, how are they separated, how do they affect each other, and where do they conflict with each other? These are questions that should also play a role in future research on other techno-fixes. Because it is only on the basis of such a script analysis that it is possible to assess which social problems a specific techno-fix actually wants to solve and what images of nature, technology and society we would have to accept for it.
